# Diffuse pulmonary hemorrhage as presenting syndrome of HIV disease

**Published:** 2010-06-18

**Authors:** Jagdish Prasad Goyal, Vijay B Shah, Nagender Kumar

**Affiliations:** 1 Department of Pediatrics, Government Medical College, Surat, Gujarat, India

**Keywords:** Pulmonary, Hemorrhage, HIV

## Abstract

Pulmonary hemorrhage is infrequently encountered in pediatrics. It can occur in isolation or in association with an underlying condition. We report a case of diffuse pulmonary hemorrhage as a presenting syndrome of HIV disease.

## Background

Pulmonary hemorrhage is infrequently encountered in pediatrics, when present it is often focal and self-limiting. Rarely bleeding reflects diffuse alveolar hemorrhage (DAH). The diagnosis of pulmonary hemosiderosis refers to this more chronic and diffuse alveolar process. Pulmonary hemosiderosis can occur in isolation or in association with an underlying condition [1]. We present a case of diffuse pulmonary hemorrhage as a presenting syndrome of HIV disease.

## Patient and case report

A 2.5 years old male child, weighing 6.5 kg presented to us with fever, cough, respiratory difficulty and generalized edema for 2 weeks. There was no history of delayed fall of cord, sib death or hemoglobinopathy in the family. On examination the child was severely anemic, having tachycardia, tachypnea and hepatosplenomegaly.

Investigations revealed Hemoglobin(Hb) 1.9 gm/dl, Total Leukocyte Count (TLC) 19.8×10^3^ /µL, Platelet Count (PC) 69×10^3^/µL, corrected reticulocyte count 4%, ESR 45 mm/ h. Renal Function Test  and Liver Function Test was normal but serum albumin was low (1.5 gm/dl). Na 136 mEq/L, K 3.1mEq/L, and Ca were 6.5 mg/dL.  An X-ray of the chest was suggestive of bilateral diffuse infiltrates. The bone marrow of the child was suggestive of iron deficiency anemia, no blast or Leishman Donovan (LD) bodies for kala-azar. ELISA for anti- HIV antibody, gastric aspirate (GA) for acid fast bacilli (AFB) was negative and Mantoux test with 5 TU was 0mm after 72 hours. Patient was started on cefotaxime with amikacin without improvement after a few days. Blood culture grew klebsiella on 5^th^ day which was sensitive to Gatifloxacillin. Consequently the child’ treatment was switched to Gatifloxacillin for 14 days. In addition the child also received antibiotics packed cell transfusion and calcium. The child responded well clinically as well as radiologically.

After 15 days the child was readmitted with fever, cough and respiratory distress. Complete blood count (CBC) showed Hb 6.5 gm/dl, TLC 8.5×10^3^ /µL, PC 240×10^3^/µL. The X- ray of the chest was suggestive of bilateral diffuse infiltrates. The child was provided with supportive management in the form of oxygen, intravenous fluid and packed cell transfusion. He improved within a week. He was again readmitted after one month with similar complaints of cough and respiratory distress. His CBC revealed Hb 4.3 gm/dl, TLC 5.2×10^3^ /µL, PC 40×10^3^/µL. The chest X-ray was again showing bilateral diffuse infiltrates. This time the child did not improve with symptomatic and supportive management in form of oxygen, intravenous fluid and packed cell transfusion even after a week. Pediatric pulmonologist opinion from a distant institute was taken and gastric aspirate (GA) was sent which showed abundant hemosiderin laden macrophage. ELISA for anti-HIV antibody was also positive this time which was confirmed by repeat test. His CD4 count was also low. IgG and IgM for CMV were negative. The patient was started on systemic steroid and hydroxychloroquine (HCQ). He responded dramatically with the treatment. He was discharged on oral iron, inhaled corticosteroid (ICS) and HCQ and planned to start antiretroviral therapy (ART) on follow up. After one month of follow up, the patient has had no recurrence of symptoms; his Hb is 9.8gm/dl and chest radiograph shows clearing haziness. Anti-retroviral therapy (ART) was started from ART centre.

## Discussion

We describe a case of diffuse pulmonary hemorrhage as presenting symptom of HIV disease based on iron deficiency anemia, diffuse alveolar infiltrates, presence of hemosiderin laden macrophage in GA and positive anti-HIV antibody. Pulmonary hemorrhage is relatively uncommon in pediatric practice. It has potentially fatal occurrences. Diffuse, slow bleeding in the lower airway may become severe and present with anemia, fatigue or respiratory compromise. Hemoptysis must always be separated from hematemesis and epistaxis, as all can present similarly in young patients [1].

The diagnosis of pulmonary hemosiderosis refers to a more chronic and diffused alveolar process. Pulmonary hemosiderosis has classically been characterized by the triad of iron deficiency anemia, hemoptysis and the alveolar infiltrates on chest radiographs. A high level of clinical suspicion is required because any or all of these features of the disease may be absent at any point of time [2]. Hemoptysis is unusual as children swallow blood stained sputum and alveolar bleeding does not readily gain access to central airways [3,4].

The management of pulmonary hemosiderosis comprises of supportive therapy including volume resuscitation, ventilator support, supplemental oxygen and transfusion of blood products. Systemic corticosteroids are the first line of treatment for the acute alveolar hemorrhage state. Other immunosuppressive agents such as hydroxychloroquine, azathioprine (AZA), cyclophosphamide and gammaglobulin can be used in children who fail to respond to corticosteroids. Picard et al observed significant improvement in clinical symptoms as well as pulmonary functions in a patient of idiopathic pulmonary hemosiderosis when treated monthly with intravenous gammaglobulin along with AZA and steroids [5]. Early treatment with corticosteroids appears to decrease the episodes of hemorrhage. This therapy may also modulate the neutrophil influx and inflammation associated with hemorrhage, thereby decreasing progression towards fibrotic disease [6].

Vincent et al [7] in their prospective study of broncho-alveolar lavage (BAL) in HIV infected patients found alveolar hemorrhage is frequently diagnosed during BAL in these patients. Presence of AH may be due to an underlying cause, such as pulmonary Kaposi Sarcoma, Cytomegalovirus pneumonia, hydrostatic pulmonary edema, or to triggering factors such as thrombocytopenia. Our patient was found to be HIV positive later on. Thrombocytopenia may be a triggering factor in our case. This case report highlights a rare combination of pulmonary hemorrhage and HIV and also underscores the importance of antigen (PCR) test for early diagnosis of HIV, as ELISA may delay the diagnosis particularly when patient is in window period.

## Conclusion

We must consider the diagnosis of pulmonary hemorrhage in patients having cough, dyspnea; iron deficiency anemia; and infiltrates on the chest radiograph. Investigation workup should be done to rule out secondary causes particularly tuberculosis and HIV in prevalent area.

## Acknowledgements

Dr. S.K. Kabra , Professor of Pediatrics, In-charge Pediatric Pulmonologist, All India Institute of Medical sciences, New Delhi for his kind suggestion during diagnosis and management of patient.

## Competing interests

The authors declared that they have no competing interests.

## Authors’ contributions

**JPG & NK:** were involved in diagnosis and management of patient. **JPG**: wrote manuscript. **VBS:** critically reviewed the manuscript
